# An Overview of Extensively Drug-resistant Salmonella Typhi from a Tertiary Care Hospital in Pakistan

**DOI:** 10.7759/cureus.5663

**Published:** 2019-09-16

**Authors:** Nadia Saeed, Muhammad Usman, Ejaz A Khan

**Affiliations:** 1 Internal Medicine, Shifa International Hospital, Shifa Tameer-E-Millat University, Islamabad, PAK; 2 Pathology, Shifa International Hospital, Shifa Tameer-E-Millat University, Islamabad, PAK; 3 Pediatrics, Shifa International Hospital, Shifa Tameer-E-Millat University, Islamabad, PAK

**Keywords:** drug resistance, extensively drug-resistant salmonella typhi, pakistan, salmonella typhi, xdr

## Abstract

Introduction: Since 2016, the province of Sindh is in the limelight because of its association with the emergence and spread of extensively drug-resistant Salmonella typhi (XDR S. typhi). Although its global spread has been proven in several studies, our information regarding its countrywide existence is still insufficient. In the last four years, few cases of XDR S. typhi were identified at the Shifa International Hospital (SIH), Islamabad, Pakistan. This article aims to report demographic patterns, clinical presentations, and treatment outcome of these cases.

Materials and methods: This study was conducted at SIH, Islamabad, on blood culture-proven XDR S. typhi cases from January 2015 to December 2018. The data were retrieved from the hospital’s record system. Patient demographic details, clinical presentations, management, and disease outcomes were evaluated and statistical analysis was performed through IBM SPSS Statistics for Windows, version 23.0 (IBM Corp., Armonk, NY).

Results: A total of 30 blood culture-proven XDR S. typhi cases were identified and 80% (24) of them were reported in 2018. The mean age at presentation was 12.8±9.6 years. Twelve (40%) patients came from Islamabad, nine (30%) from Rawalpindi, and eight (26.6%) from Khyber Pakhtunkhwa (KPK). All patients, except one, were prescribed meropenem and azithromycin. Three patients developed complications but no mortality was documented. Over four years, these XDR S. typhi cases contributed 5.01% to the total S. typhi isolates.

Conclusion: This study validates the existence of XDR S. typhi all over Pakistan. It stresses upon the fact that more stringent methods should be adopted for its identification and control.

## Introduction

Extensively drug-resistant Salmonella typhi (XDR S. typhi) has become one of the most pressing health issues in Pakistan. S. typhi bacteria are labeled as XDR S. typhi when they exhibit resistance to fluoroquinolones, chloramphenicol, ampicillin, trimethoprim-sulfamethoxazole, and third-generation cephalosporins [1]. The first outbreak of ceftriaxone-resistant Salmonella was identified in two sub-districts of Hyderabad, Sindh, in 2016 [2]. The genome sequencing analysis of these resistant isolates were associated with H58 haplotype. It had numerous resistant determinants including qnrS fluoroquinolones resistance gene and extended-spectrum β-lactamase blaCTX- M-15 genes, making it non-susceptible to fluoroquinolones and ceftriaxone. This clone carried a significant tendency for global spread and was capable of transforming multi drug-resistant (MDR) to XDR strains [3]. The two main treatment options for this resistant infection are meropenem and azithromycin, although, a case of azithromycin resistance has been documented from India [4-5].

According to the World Health Organization (WHO), from 2016 to December 2018, the Provincial Disease Surveillance and Response Unit (PDSRU) reported 5274 XDR S. typhi cases from 14 districts of Sindh. These included 76% cases from Karachi city, 27% cases from Hyderabad district and 4% cases from other districts of Sindh [1,6]. Despite the control measures initiated by the local government, there was a marked increase in reported cases from 2017 to 2018 [1]. The international surveillance for XDR S. typhi identified a case from the UK and five cases from the USA [7]. Recently, another case has been reported from Canada [8]. All these patients had a history of travel to Pakistan [1,7-8].

We still have inadequate information regarding the prevalence of XDR S. typhi in our country. The local literature review reveals limited case reports from areas other than the Southeastern located province, Sindh [1]. At Shifa International Hospital (SIH), Islamabad, few cases of XDR S. typhi were recognized in the last four years. SIH is a tertiary care teaching hospital located in the capital city of Pakistan. It covers a large catchment area of Northern parts of Pakistan, including North Punjab, Kashmir, Gilgit-Baltistan, and Hazara divisions. In addition, it also entertains the referrals from the province, Khyber Pakhtunkhwa (KPK).

This study was planned to report these blood culture-proven XDR S. typhi cases along with their demographic overview and treatment experiences. It also highlights the antimicrobial susceptibility patterns of S. typhi, signifying the percentage contribution made by XDR S. typhi isolates, over the study period at our hospital.

## Materials and methods

This retrospective study was conducted at SIH, Islamabad, on blood culture-proven XDR S. typhi cases from January 2015 to December 2018. The blood cultures of suspected patients were processed by the BACTEC 9240 automated system (Becton Dickinson, USA) or BACT/ALERT 3D system (Biomerieux, France). A positive blood culture showing gram-negative rods was cultured on blood and MacConkey agar plates. After overnight incubation, the growth characteristics were studied. The organisms were identified as S. typhi with the Vitek 2 system (Biomerieux, France) and confirmed serologically by S. typhi polyvalent antiserum (Bio-Rad, France). Antimicrobial susceptibility testing of the isolates was done by the disk diffusion technique and zones of inhibition were interpreted according to the Clinical & Laboratory Standards Institute (CLSI) guidelines [9]. The isolates were tested for ampicillin, co-trimoxazole, chloramphenicol, ciprofloxacin, ceftriaxone, and azithromycin susceptibility. Extended-spectrum beta-lactamases (ESBL) production was detected by the disk diffusion method and phenotypic confirmation was done by using both cefotaxime and ceftazidime alone, and in combination with clavulanate [9].

Ethics committee approval was taken from the institutional IRB. The data regarding antimicrobial susceptibility patterns of S. typhi were retrieved from the hospitals’ microbiology lab. Charts and files of XDR S. typhi cases were viewed for patient demographic details, clinical presentations, management and, outcomes of disease. Mean and standard deviations were measured for age and duration of illness. Frequencies and percentages were calculated for gender, clinical characteristics and residence by using IBM SPSS Statistics for Windows, version 23.0 (IBM Corp., Armonk, NY).

## Results

From January 2015 to December 2018, a total of 30 cases with blood culture-proven XDR S. typhi, were identified at SIH, Islamabad. All the isolates were extended-spectrum β-lactamase producers and were sensitive to meropenem and azithromycin only.

Among these cases, 24 (80%) patients were male while females were only six (20%). The mean age at presentation was 12.8±9.6 years, with the minimum and maximum limits between two to 36.5 years. Table 1 demonstrates the age distribution at the time of presentation, indicating that 21 (70%) patients were less than 15 years old.

**Table 1:** Age distribution of patients with extensively drug-resistant Salmonella typhi (n=30)

Age at the time of presentation	Frequency of patients	Percentage of patients
< 5 years	5	16.7 %
5 to 10 years	10	33.3 %
>10 to 15 years	6	20 %
>15 years to 20 years	3	10 %
>20 to 30 years	3	10 %
>30 years	3	10 %
Total	30	100 %

Twelve (40%) patients were identified from Islamabad, nine (30%) from Rawalpindi (North Punjab), eight (26.6%) from KPK and one (3.33%) from Wah Cantonment (North Punjab). Six out of 12 patients from Islamabad belonged to central and well-developed areas of the city. Two patients from Islamabad had recently traveled to Karachi and another two had frequent travel history to KPK.

The first blood culture-proven case of XDR S. typhi was detected in March 2015. He was a 14-year-old boy, residing in Satellite town at Rawalpindi City. Later in 2015, three more cases were identified, two in July and one in September. All these cases belonged to Islamabad and Rawalpindi. We had one case each in 2016 (from KPK) and 2017 (from Islamabad), and then in 2018, 24 patients with XDR S. typhi were identified from Rawalpindi, KPK, Wah Cantonment, and Islamabad.

All the patients had a high-grade fever of 6.8±1.1 days. Sore throat, and vomiting and loose motions were found in 15 (30%) and 10 (33.3%) patients, respectively. Many patients had taken antibiotics including cefixime (50%), ceftriaxone (26.6%) and azithromycin (11.7%), before arrival to SIH. Eleven (36.6%) patients refused admission and managed antibiotics at their vicinity, while the rest were admitted. Few patients developed complications. An adult (20-year-old male), presented in the emergency with septic shock, and developed acute hepatitis and acute respiratory distress syndrome during the course of illness. However, he responded well to meropenem and azithromycin and was discharged in stable condition. Another two-year-old boy developed typhoid meningitis, but he also recovered after treatment. A 26-year-old male had ileal perforation during the hospital stay. He underwent exploratory laparotomy, small bowel resection and, ileostomy, and went home in healthy condition. Considering severe sepsis, all the above-mentioned patients and two others were given dexamethasone, in addition to antibiotics.

All cases except one were prescribed meropenem and azithromycin. One patient was treated with azithromycin and he improved. No mortality was documented.

At SIH, from January 2015 to December 2018, enteric fever causing Salmonellae were isolated from the blood cultures of 832 patients. Amongst these, 598 (71.85%) were identified as S. typhi. Of 598 S. typhi cases, 375 (62.7%) were MDR and 30 (5.01%) were XDR [1]. We did not identify any resistant Salmonella paratyphi case in these four years. The yearly antimicrobial susceptibility pattern of S. typhi is shown in Table 2.

**Table 2:** Characteristics of blood culture-proven Salmonella typhi isolates from 2015 to 2018 (n=598) Multi drug-resistant Salmonella typhi: resistant to chloramphenicol, ampicillin, and trimethoprim-sulfamethoxazole; Extensively drug-resistant Salmonella typhi: resistant to fluoroquinolones, chloramphenicol, ampicillin, trimethoprim-sulfamethoxazole, and third-generation cephalosporins.

Year	Total Salmonella Typhi isolates n (%)	Multi drug-resistant Salmonella Typhi n (%)	Extensively drug-resistant Salmonella Typhi n (%)
2015	163 (27.25%)	106 (17.72%)	4 (0.66%)
2016	119 (19.9%)	77 (12.87%)	1 (0.16%)
2017	175 (29.26%)	108 (18.06%)	1 (0.16%)
2018	141 (23.57%)	84 (14%)	24 (4.01%)
Total cases	598 (100%)	375 (62.7%)	30 (5.01%)

## Discussion

After the identification of the XDR S. typhi from Hyderabad, local surveillance was initiated which led to the identification of thousands of cases from Sindh [2]. International surveillance also identified a few cases [7]. However, apart from Sindh, there is scarcity in the documentation of cases from Pakistan. Our extensive data search from local literature revealed very few XDR S. typhi cases. The first case of extended-spectrum β-lactamase producing S. typhi was reported from Social Security Hospital, Lahore (Punjab) in 2012 [10]. The second case reported was from Rawalpindi in 2016 [11]. In addition, according to WHO, two international travel-related XDR S. typhi cases had a history of travel to Lahore and Islamabad [1]. 

Several studies have proven the presence of MDR S. typhi all over Pakistan. MDR S. typhi are resistant to the first-line antibiotics, which are chloramphenicol, ampicillin, and trimethoprim-sulfamethoxazole [1]. A recent study from Islamabad in 2017 indicated that out of 197 patients with typhoid fever, 64 S. typhi and five S. Paratyphi isolates were MDR, however, no resistance to cefixime and ceftriaxone was identified [12]. Another retrospective multicenter study about the identification of antibiotic resistance pattern on blood culture-proven typhoid cases, from three tertiary care hospitals of Pakistan, included cases from Armed Forces Institute of Pathology (AFIP), Rawalpindi. The data from 2005 to 2014 demonstrated a high burden of fluoroquinolone-resistant and MDR S. typhi strains. However, this study, also, did not report any XDR S. typhi case from AFIP [13]. 

The capital city Islamabad is known for its relatively better health, water and, sanitation facilities. The areas located at the periphery of Islamabad, however, lack basic facilities. Presence of XDR S. typhi in and around Islamabad is alarming. Our study indicates that XDR S. typhi existed in this city in 2015, prior to the first documented case from Sindh in November 2016 [2]. The record review of this patient did not reveal any travel history outside Islamabad. In 2016 and 2017, the number remained low, but the sudden upsurge of new cases in 2018 indicates that the northern areas of Pakistan are also heading towards this catastrophe. Although apparently the spread and expansion of this disease is not comparable to Sindh, it can be a secondary to under-reporting of cases. We also had eight inhabitants of KPK province. To our knowledge, until now, XDR S. typhi reports from KPK are non-existing.

The age distribution of our effected patients was dominated by children, as is also observed in other reports from Pakistan [1-2,7]. The prominent male predisposition directs that the determinants for the incidence of XDR S. typhi should be explored.

Contaminated water and food, inadequate hygiene and sanitation measures, use of untreated sewage for crop fertilizer are important sources for typhoid transmission [14]. Few local research articles attributed the emergence and spread of XDR S. typhi to the alarming sewage and water systems, low vaccination rates, injudicious antibiotic use, and overpopulated city dwellings in Sindh [15]. The conditions of sewage and water systems in the northern areas of Pakistan are not different from Sindh. Islamabad and Rawalpindi are the two major cities, using surface water for drinking purposes. The unprocessed surface water is not safe for drinking as it contains raw municipal and industrial effluents and agriculture runoff [16]. A study revealed that the water from Rawal Lake and its distributions channels, which are the main drinking water source for residents of Islamabad and Rawalpindi, was highly contaminated with bacteria [17]. Similarly, drinking water analysis from Peshawar and Swat (KPK) demonstrated fecal contamination [18-19]. 

In addition to the water and sanitation issues, several studies have confirmed the presence of MDR and XDR-Salmonella serovars in poultry and other farm animals [20-21]. The injudicious use of antibiotics in farm animals is contributing to the development of antibiotic-resistant genes in foodborne pathogens [22]. A local research on the prevalence of Salmonella typhimurium and Salmonella enteritidis serovars in poultry, concluded that all isolates of Salmonella were MDR and 8.8% were extended-spectrum beta-lactamase producers and had zoonotic potential [23]. Another study from Islamabad and northern Punjab reported a high prevalence of Salmonella isolates in red meat, fish, chicken organs, eggs, and their feed contents [24]. 

The Government of Pakistan has taken several steps including awareness campaigns about sanitation, hygiene, water purification and rational use of antibiotics. More than 118,000 children of age between six months to ten years have been vaccinated with typhoid conjugate vaccine (Typbar-TCV®), in Hyderabad [1]. Typbar-TCV® was prequalified by WHO in 2018 due to its better efficacy, long-lasting immunity and ability to be administered to young children of six months [25]. A national task force was formed which is working in conjunction with WHO and United States Centers for Disease Control and Prevention to draft a national action plan to contain this outbreak.

The considerable increase in XDR S. typhi cases directs that immediate preventive and control measures should be instituted at all levels. It includes the extension of strict surveillance programs all over the country. We need a mass media campaign to enhance awareness about the importance of cleanliness and hygiene, including adequate hand washing. The government needs to take comprehensive steps to provide clean and safe drinking water, to arrange for effective and sanitary disposal of human feces and urine, and to enforce regulations for manufacturers of food products and beverages to ensure food safety. The exponential rise in the number of XDR S. typhi cases demands special attention for the provision of better health care facilities, establishment of affordable and efficient laboratory networks, and widespread availability of Typbar-TCV®. Vigilance and accountability are required to control antibiotics misuse in humans and animals. We also need to identify the prevalence of chronic carriers of Salmonella, which might be an important source for typhoid transmission. 

To our knowledge, these are the largest case numbers reported from any hospital in the northern areas of Pakistan. We did not have any missing data and files of all patients were analyzed for the study. There are several limitations to our study. Since it involved records review, we were not able to explore the travel histories, food and drinking water resources, and vaccination status of our patients. Also, it was a single-center study. Representative data from other hospitals, especially from primary health care facilities of Islamabad, should be evaluated to draw more accurate conclusions. Furthermore, we did not assess the genetic characteristics of our resistant isolates which could have provided more insight about their resistance determinants.

## Conclusions

This study underscores the presence of XDR S. typhi in and around Islamabad and combined with other local studies, we can conclude their existence all over Pakistan. Surveillance studies are required at all levels, for risk stratification and control of this disease. Our study also signifies the role of preventive measures, better health care facilities, antibiotic stewardship, and vaccination, at a national level, to restrict this menace.
